# Glycemic Variability and Continuous Glucose Monitoring in Occupational Health: A Narrative Review of Emerging Evidence and Potential Applications in Working Populations

**DOI:** 10.3390/healthcare14131979

**Published:** 2026-07-03

**Authors:** Aikaterini Andreadi, Stella Andreadi, Federica Todaro, Marco Cerilli, Pietro Lodeserto, Giuseppe Pinto, Marco Meloni, Alfonso Bellia, Luca Coppeta, Andrea Magrini, George P. Chrousos, Davide Lauro

**Affiliations:** 1 Section of Endocrinology and Metabolic Diseases, Department of Systems Medicine, University of Rome Tor Vergata, 00133 Rome, Italy; 2 Endocrinology and Diabetology Clinic, Department of Medical Sciences, Foundation Policlinico Tor Vergata, 00133 Rome, Italy; 3 Department of Biomedicine and Prevention, University of Rome Tor Vergata, 00133 Rome, Italy; 4 University Research Institute of Maternal and Child Health and Precision Medicine, Medical School, National and Kapodistrian University of Athens, 11527 Athens, Greece; 5 United Nations Educational, Scientific and Cultural Organization (UNESCO) Chair on Adolescent Health Care, 11527 Athens, Greece; 6 University Research Institute, Choremeion Bldg., Aghia Sophia Children’s Hospital, 11527 Athens, Greece

**Keywords:** glycemic variability, occupational health, continuous glucose monitoring, worker health surveillance

## Abstract

Background: Fasting plasma glucose, glycated hemoglobin (HbA1c), and oral glucose tolerance testing remain central to the diagnosis and monitoring of dysglycemia, but they mainly reflect the average glycemic exposure or discrete time-point measurements and may not capture intraday and interday glucose fluctuations. Glycemic variability (GV) has been associated with oxidative stress, endothelial dysfunction, inflammation, and diabetes-related complications, although much of the evidence derives from experimental, clinical, and diabetes-care settings rather than occupational cohorts. Aim: This narrative review examines the physiological basis, measurement, and potential occupational relevance of GV and continuous glucose monitoring (CGM) in working populations. Methods: Literature was narratively selected from biomedical databases, major guidelines, consensus statements, and occupational-health sources, prioritizing reviews, clinical guidelines, cohort studies, mechanistic studies, and CGM studies. No systematic search, risk-of-bias assessment, or quantitative synthesis was performed. Main findings: CGM is an established technology in selected diabetes-care contexts and provides metrics such as coefficient of variation, time in range, time above range, and time below range. Its use in occupational medicine, however, remains investigational outside selected clinical circumstances. Work-related factors such as shift work, circadian disruption, sleep loss, psychosocial stress, irregular meal timing, sedentary behavior, and variable physical workload may influence glucose regulation, but direct evidence linking these exposures to CGM-measured GV in workers remains limited. Implications: Potential applications include research on occupational determinants of metabolic health, monitoring of workplace lifestyle interventions, and individualized management of workers with diabetes in safety-sensitive roles, provided that consent, confidentiality, clinical follow-up, equity, and data-governance safeguards are ensured. Conclusions: GV assessment may complement traditional metabolic markers in selected occupational-health contexts, but routine CGM-based surveillance of general worker populations is not currently supported by sufficient evidence. Further longitudinal and interventional studies are required.

## 1. Introduction

Type 2 diabetes, prediabetes, obesity, and insulin resistance are major concerns for working-age populations. The 11th edition of the International Diabetes Federation Diabetes Atlas estimates that approximately 589 million adults aged 20–79 years were living with diabetes in 2024, with prevalence projected to continue increasing [1]. Because many affected individuals are active members of the workforce, metabolic health is relevant not only to long-term morbidity, but also to occupational prevention, job adaptation, and safe performance in selected safety-sensitive tasks.

Occupational health surveillance commonly relies on static biochemical markers such as fasting plasma glucose (FPG), glycated hemoglobin (HbA1c), and the oral glucose tolerance test (OGTT). These tests are central to the diagnosis and classification of diabetes and prediabetes according to current international guidance [2,3]. However, they provide limited information about short-term glucose dynamics during work shifts, sleep periods, irregular meals, or variable physical workload. This distinction is particularly important in occupational settings where circadian disruption, sleep loss, psychosocial stress, and meal timing may alter glucose regulation across the day.

Glycemic variability (GV) refers to intraday and interday fluctuations in glucose concentration. In diabetes-care and mechanistic studies, GV has been associated with oxidative stress, endothelial dysfunction, inflammatory activation, and adverse outcomes [4,5,6,7]. These associations are clinically relevant, but they should not be interpreted as proof that GV independently causes disease in all populations. Evidence in general working populations remains limited, and thresholds developed for diabetes care cannot be automatically transferred to metabolically healthy workers or occupational screening programs.

Continuous glucose monitoring (CGM) has made it possible to characterize glucose profiles under real-world conditions. CGM is established for selected people with diabetes and provides indices such as standard deviation (SD), coefficient of variation (CV), mean amplitude of glycemic excursions (MAGE), time in range (TIR), time above range (TAR), and time below range (TBR) [8,9,10]. In occupational medicine, however, CGM remains an emerging research and targeted clinical concept rather than a routine surveillance tool.

The main knowledge gaps are the scarcity of direct occupational CGM studies, the absence of validated GV thresholds for workers without diabetes, limited longitudinal evidence linking workplace exposures to CGM-derived metrics and future metabolic disease, and insufficient ethical guidance for the workplace use of wearable metabolic data. This narrative review therefore examines the physiological basis and measurement of GV, the strengths and limitations of CGM technologies, the available occupational evidence, and the practical, ethical, and research implications for occupational physicians.

Targeted searches were conducted in PubMed/MEDLINE and Google Scholar and were supplemented by official guideline repositories and the manual screening of reference lists from key reviews, consensus reports, and occupational-health publications. Searches covered records available through 21 June 2026, with no predefined lower date limit. Search concepts combined terms related to glycemic variability, continuous glucose monitoring, fasting plasma glucose, HbA1c, oral glucose tolerance testing, endothelial or microvascular dysfunction, shift work, night work, circadian disruption, sleep deprivation, psychosocial stress, occupational medicine, workplace health surveillance, wearable technology, and data privacy.

Priority was given to current guidelines and consensus statements, systematic reviews, large cohort studies, mechanistic studies, direct CGM studies, and publications with explicit occupational relevance. Recent evidence was prioritized for CGM technologies and occupational applications, whereas earlier landmark studies were retained when foundational. No protocol was registered, and duplicate screening, formal risk-of-bias assessment, meta-analysis, and quantitative evidence grading were not performed. The findings should therefore be interpreted as a critical narrative synthesis rather than an exhaustive systematic review.

## 2. Glycemia as a Marker of Metabolic Risk

FPG, HbA1c, and OGTT remain the foundation for the diagnosis and monitoring of dysglycemia. FPG reflects basal glucose regulation after fasting, OGTT evaluates the response to a standardized glucose challenge, and HbA1c reflects the average glycemic exposure over approximately two to three months, with greater weighting toward the most recent weeks [2,3,11]. These markers are clinically validated, widely available, and appropriate for periodic health examinations when used according to clinical guidelines.

### 2.1. Traditional Glycemic Markers

Fasting plasma glucose (FPG), glycated hemoglobin (HbA1c), and the oral glucose tolerance test (OGTT) are the most commonly used lab tests for assessing blood sugar levels. These tests form the basis of the current diagnostic criteria for diabetes and prediabetes, as outlined in international clinical guidelines.

FPG is a widely used screening and diagnostic measure of glucose metabolism. It is measured after an overnight fast and reflects basal glucose regulation; current diagnostic thresholds should be interpreted according to clinical guidelines [2,3].

HbA1c is a cornerstone of metabolic assessment and reflects the average glycemic exposure over the preceding two to three months, with greater weighting toward recent weeks [11]. Its interpretation can be affected by conditions that alter erythrocyte turnover or hemoglobin structure.

The OGTT evaluates plasma glucose before and after a standardized glucose load and is particularly useful for identifying impaired glucose tolerance [2,3].

Together, these markers identify impaired fasting glucose, impaired glucose tolerance, and overt diabetes, conditions associated with microvascular and macrovascular complications [12].

FPG, HbA1c, and OGTT remain the foundation of dysglycemia diagnosis and monitoring. Their occupational relevance lies primarily in detecting established metabolic risk rather than characterizing glucose dynamics during individual work shifts.

Their occupational relevance lies in detecting established metabolic risk factors rather than in capturing the workday glucose dynamics. A worker with a normal FPG may still experience postprandial excursions during night work, and two individuals with similar HbA1c values may have different patterns of hypoglycemia, hyperglycemia, and GV. Conversely, isolated abnormal CGM patterns in a person without diabetes do not necessarily indicate disease and require careful clinical interpretation.

### 2.2. Limitations of Static Glycemic Indicators

While traditional glycemic markers are clinically useful, they have limitations when used alone to assess metabolic control. A key limitation is that these markers primarily reflect the average blood sugar levels rather than daily glucose fluctuations.

HbA1c reflects the average glycemic exposure but does not directly identify daily glucose fluctuations or episodes of hypoglycemia and hyperglycemia. Consequently, two people with comparable HbA1c values may have markedly different glucose profiles [13].

Likewise, fasting plasma glucose, a snapshot measurement, may not accurately reflect glycemic dynamics during postprandial phases, where considerable glucose excursions are often observed.

Another limitation stems from biological and clinical factors that affect interpretation. Altered erythrocyte turnover, anemia, hemoglobin variants, chronic kidney disease, pregnancy, recent blood loss or transfusion, and iron deficiency may alter HbA1c independently of glucose exposure [14]. Acute illness, stress, and sleep loss may also affect fasting glucose.

Experimental and clinical evidence suggests that intermittent hyperglycemia may activate oxidative and inflammatory pathways beyond mean glucose exposure in selected contexts [5,6,15]. This evidence derives mainly from diabetes-care and mechanistic studies and should not be generalized uncritically to workers without diabetes.

These limitations do not reduce the diagnostic importance of traditional markers; rather, they define the situations in which dynamic measures could provide complementary information. Table 1 summarizes the main limitations and occupational implications of static indicators.

### 2.3. Glycemia, Endothelial Function, and Microvascular Dysfunction

Beyond their diagnostic role, hyperglycemia and postprandial glucose excursions have been associated with endothelial and microvascular dysfunction through reduced nitric oxide bioavailability, oxidative stress, inflammatory signaling, and prothrombotic pathways [5,6,7,15].

Recent studies using laser speckle contrast analysis have reported altered skin microvascular reactivity in prediabetes and type 2 diabetes and associations between glucose measures and microvascular dysfunction [16,17]. Most of this evidence is cross-sectional or mechanistic; it should not be interpreted as proof that GV independently causes microvascular dysfunction in working populations. Recent microvascular studies using laser speckle contrast analysis (LASCA) suggest that prediabetes and type 2 diabetes are associated with impaired skin microvascular reactivity and that glucose levels may be independently related to microvascular dysfunction [16,17]. These findings support the concept that glucose regulation may influence vascular function before overt clinical complications develop. However, the available evidence is primarily cross-sectional or mechanistic and does not prove that GV independently causes microvascular dysfunction in working populations.

## 3. Understanding Glycemic Variability

**Figure 1:**
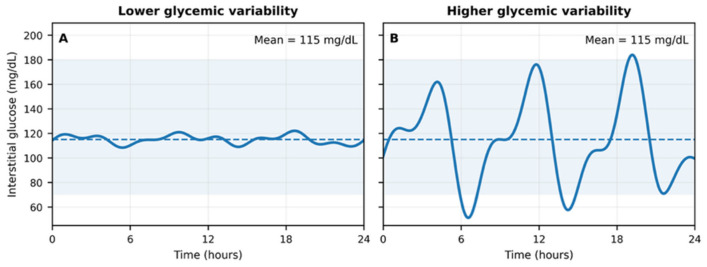
(**A**,**B**) Conceptual representation of glycemic variability compared with stable glycemic control: Conceptual 24-h interstitial glucose profiles with the same mean glucose (115 mg/dL) but different variability. The shaded band (70–180 mg/dL) is included only as an illustrative adult diabetes-care range; it is not a validated threshold for occupational screening. Curves are schematic and do not represent patient data. This schematic diagram illustrates the difference between stable glycemic control and high glycemic variability. In both scenarios, the mean glucose level is similar; however, glucose dynamics differ substantially. In the stable pattern, glucose concentrations remain relatively constant over time, whereas high glycemic variability is characterized by repeated fluctuations between hyperglycemia and hypoglycemia. These oscillations may occur despite similar average glucose values and may contribute to additional metabolic risk.

In recent years, the concept of glycemic variability (GV) has gained increasing attention as an important aspect of glucose regulation. While traditional measures like HbA1c provide a snapshot of average glucose exposure, they do not fully capture the degree or frequency of daily glucose fluctuations. Glycemic variability, the degree of change in blood glucose levels over time, is a dynamic aspect of metabolic control with unique clinical implications. The concept of glycemic variability compared with stable glycemic control is illustrated in Figure 1.

### 3.1. Definition and Physiological Basis

Glycemic variability includes short-term intraday and longer-term interday changes in glucose concentration. Its measurement depends on the data source, sampling frequency, observation duration, and selected metric; therefore, GV is not a single uniform construct [13,15,18].

Glucose homeostasis reflects the interaction among pancreatic beta-cell function, insulin secretion and sensitivity, hepatic glucose production, incretin effects, counter-regulatory hormones, sleep, meal timing, diet composition, and physical activity [13,18,19]. In insulin resistance, prediabetes, or diabetes, these regulatory systems may become less efficient, resulting in larger postprandial excursions, and in treated diabetes, potential hypoglycemic events.

Postprandial glucose excursions may contribute substantially to overall glycemic exposure, even when fasting glucose appears acceptable [19].

### 3.2. Metrics of Glycemic Variability

The increasing adoption of continuous glucose monitoring has spurred the development of numerous numerical indices to evaluate glycemic variability. These indices are intended to characterize distinct facets of glucose dynamics, encompassing the magnitude, periodicity, and persistence of blood sugar oscillations.

One of the most commonly used indicators is the standard deviation (SD) of glucose values, which reflects the dispersion of glucose measurements around the mean glucose level. However, SD alone may be influenced by the average glucose value and therefore may not accurately reflect relative variability.

The coefficient of variation (CV), calculated as the standard deviation divided by mean glucose and expressed as a percentage, is a commonly used relative variability measure. A CV threshold of approximately 36% is derived from diabetes-care consensus recommendations and should not be generalized to workers without diabetes or to occupational screening [8,20].

Mean amplitude of glycemic excursions (MAGE) quantifies the average magnitude of major glucose excursions between peaks and nadirs [21]. It is calculation-dependent, less intuitive than CV, and sensitive to data quality and analytical method.

Time in range (TIR) is the percentage of time spent within a predefined glucose range. The frequently used range of 70–180 mg/dL is intended for many adults with diabetes and is not a universal threshold for healthy workers, people with prediabetes, pregnancy, or specific safety-sensitive contexts [8,22].

Together, these metrics provide complementary information on glucose dynamics and enable clinicians and researchers to better characterize patterns of glycemic instability that may not be evident in traditional laboratory measurements, as summarized in Table 2.

### 3.3. Pathophysiological Implications

Growing evidence suggests that glycemic variability can independently harm metabolism and blood vessels, even when blood sugar levels are consistently high. Several experimental studies have shown that sudden changes in glucose can start oxidative stress pathways. This leads to an increase in reactive oxygen species, which then causes cellular damage.

Experimental and clinical evidence suggests that intermittent hyperglycemia can activate oxidative and inflammatory pathways beyond mean glucose exposure in selected contexts [5,6,7,15]. Proposed mechanisms include mitochondrial reactive oxygen species generation, activation of protein kinase C and advanced glycation pathways, reduced nitric oxide bioavailability, endothelial dysfunction, and inflammatory signaling.

Human observational studies and clinical reviews have associated higher GV with adverse outcomes in diabetes and critically ill populations [4,13,23,24]. These findings should be interpreted cautiously because they do not establish the same prognostic value in workers without diabetes, and confounding by baseline glycemia, medication use, acute illness, diet, or sleep may contribute to observed relationships.

Experimental studies indicate that oscillating glucose may provoke greater oxidative and endothelial responses than sustained hyperglycemia under selected conditions [5,7].

Glucose fluctuations have been linked to inflammatory signaling, reduced nitric oxide bioavailability, and endothelial dysfunction [6,7,15]. These mechanisms are relevant to diabetic vascular complications, but direct causal evidence in occupational cohorts is lacking.

Clinical studies suggest that higher GV may correlate with adverse outcomes in selected patient populations, particularly in critical illness [4,23,24]. The causal and prognostic implications outside these contexts remain uncertain.

Glycemic variability, a concept that enhances our understanding of glucose regulation, could provide crucial insights beyond traditional measures of glycemic control. With advances in monitoring technologies, the assessment of glycemic variability is set to become increasingly important in clinical research and the evaluation of metabolic risk.

## 4. Technologies for Assessing Glycemic Variability

Advancements in modern glucose monitoring technologies have improved how we assess glycemic variability. Traditionally, self-monitoring of blood glucose (SMBG), which involves using portable glucometers to test capillary blood, was the main method for daily glucose monitoring in people with diabetes. Although SMBG provides useful information about glucose levels, its ability to fully capture the dynamic nature of glucose fluctuations is limited because measurements are usually taken only a few times a day. As a result, significant glycemic events, especially nighttime hypoglycemia or post-meal spikes, may go unnoticed.

Advances in sensor technology have substantially improved the ability to observe glucose patterns over time. CGM systems measure interstitial rather than plasma glucose, usually at short intervals, and interpretation should account for physiological lag and reduced accuracy during rapid glucose change [10,25].

CGM systems generally include a subcutaneous sensor, a transmitter, and a receiver or smartphone application. They provide glucose trends and support the calculation of CGM-derived exposure and variability metrics [10,25].

CGM is widely used in diabetes care and can improve glycemic outcomes and reduce hypoglycemia in selected populations when integrated into individualized treatment [10,26].

Two main types of CGM systems are real-time CGM (rtCGM) and intermittently scanned CGM (isCGM). rtCGM continuously transmits readings and may provide alarms, whereas isCGM requires scanning to display values, although the sensor records data continuously [10,27]. The principal technologies are summarized in Table 3.

Both technologies have greatly expanded the options for measuring glycemic variability in research and clinical practice. Besides traditional diabetes management, CGM is increasingly used in metabolic research to better understand how glucose is regulated in different physiological and pathological states.

Beyond clinical diabetes management, digital health and wearable technologies have stimulated interest in less-invasive glucose sensing [28]. However, many non-invasive consumer devices remain investigational; devices that claim to estimate glucose without penetrating the skin should not guide clinical or occupational decisions unless they are appropriately validated and authorized [29].

These advancements could be especially pertinent to occupational health research, given that understanding the interplay between work-related factors and metabolic regulation requires instruments capable of documenting physiological alterations across daily routines. Continuous monitoring technologies present the opportunity to assess glucose responses to work schedules, physical exertion, stress exposure, and sleep disturbances within authentic, everyday contexts.

As these technologies become more accessible and reliable, they could provide new methods to study metabolic processes in working populations. This would enable a more comprehensive assessment of metabolic risk, going beyond traditional lab tests.

**Table 3:** Technologies for assessing glucose dynamics and glycemic variability. Abbreviations: CGM, continuous glucose monitoring; isCGM, intermittently scanned continuous glucose monitoring; rtCGM, real-time continuous glucose monitoring; SMBG, self-monitoring of blood glucose.

Technology	Description	Strengths	Limitations/Occupational Cautions
SMBG	Capillary finger-stick measurements.	Low cost, widely available, familiar in diabetes care.	Sparse sampling; may miss nocturnal and postprandial events; user burden.
rtCGM	Continuously displays interstitial glucose and trend arrows; alarms may be available.	Real-time alerts and detailed glucose profiles.	Cost, alarm burden, false alarms, sensor lag, accuracy limits, data overload, skin irritation, and privacy.
isCGM/flash	Sensor records continuously but requires scanning for displayed values.	Lower alarm burden; useful retrospective profiles.	Missed data if not scanned; less suitable when real-time alerts are required.
Professional/blinded CGM	Clinician-applied short-term monitoring, often blinded to the wearer.	Useful for research, targeted assessment, and pattern recognition.	Requires clinical interpretation and follow-up; not appropriate for employer monitoring.
Implantable CGM	Long-duration sensor implanted subcutaneously.	Long wear period in selected diabetes-care contexts.	Procedure, cost, and device-specific calibration or backup requirements.
Non-invasive/consumer claims	Smartwatches, rings, or experimental biosensors claiming non-invasive glucose estimation.	Research interest only when scientifically validated.	Many remain unvalidated or unauthorized and should not guide decisions [29].

### Ethics, Privacy, and Governance

Any use of CGM or wearable metabolic data in occupational settings requires strict ethical and legal safeguards. CGM data are sensitive health information and should be collected only for a clearly defined clinical, preventive, or research purpose, with voluntary informed consent, data minimization, medical confidentiality, secure storage, transparent retention rules, and clear accountability [30,31].

Employers should not receive identifiable CGM traces or use glucose data to monitor productivity, discipline employees, rank performance, or make employment decisions. Interpretation should remain under the responsibility of qualified clinicians, with referral to primary care or endocrinology when clinically relevant abnormalities are detected [30,31].

## 5. Relevance in Occupational Health Practice

Metabolic health significantly influences employees’ well-being, productivity, and long-term health. In occupational medicine, surveillance initiatives aim to identify health issues that could compromise an individual’s ability to work safely and efficiently. Metabolic disorders, including obesity, insulin resistance, prediabetes, and type 2 diabetes, are becoming more common in working populations globally, and they are of particular concern. Consequently, the increasing prevalence of metabolic diseases has led occupational health professionals to explore novel strategies for early identification and risk assessment.

### 5.1. Metabolic Health in Workers

Metabolic risk in working-age adults is influenced by lifestyle, aging, and occupational exposures. Recent meta-analytic and large worker-cohort evidence supports associations between night or shift work and incident type 2 diabetes or insulin-resistance markers [32,33].

Metabolic dysfunction may affect occupational performance through fatigue, impaired concentration, and cognitive slowing. In safety-sensitive tasks, the relevant issue is the individualized risk of severe hypoglycemia or hyperglycemia, treatment regimen, awareness, and monitoring rather than the diagnosis of diabetes itself [34].

Consequently, workplace health assessments often include evaluating metabolic indicators, such as body mass index, blood pressure, lipid profiles, and fasting glucose levels. These assessments allow occupational physicians to identify employees at higher risk for cardiometabolic issues. This enables the implementation of preventive measures aimed at slowing disease progression and its related complications.

### 5.2. Glycemic Variability and Work-Related Factors

**Figure 2:**
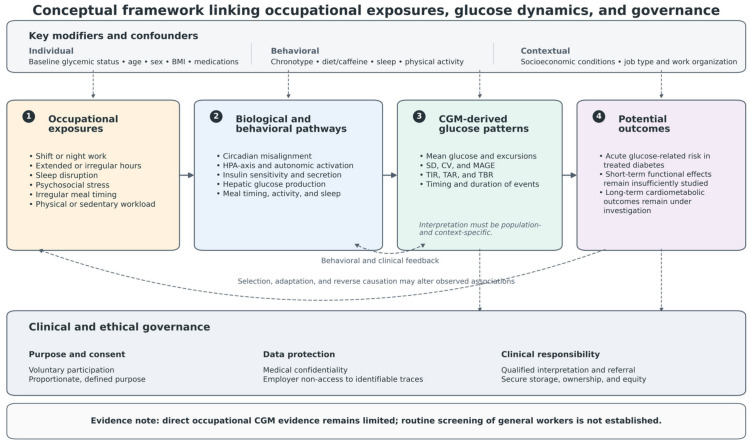
Potential interaction between occupational factors and glycemic variability. This schematic model illustrates how occupational factors may influence glucose regulation and contribute to increased glycemic variability. Work-related exposures such as shift work, sleep disruption, psychosocial stress, irregular meal timing, and variations in physical workload may alter circadian rhythms and metabolic regulation. These alterations can lead to increased fluctuations in blood glucose levels, resulting in greater glycemic variability. Over time, such metabolic instability may contribute to increased cardiometabolic risk. Understanding these interactions may support the integration of metabolic monitoring strategies, including continuous glucose monitoring, into occupational health surveillance programs.

In addition to traditional metabolic indicators, work-related factors may influence daily glucose regulation. Evidence spans diabetes-risk epidemiology, controlled physiological studies, direct CGM studies, workplace feasibility research, sleep and stress physiology, and physical-activity guidance [32,33]. Table 4 distinguishes representative evidence types, and Figure 2 summarizes the proposed interactions.

Night-shift work is associated with incident type 2 diabetes: a 2024 meta-analysis of 10 cohort studies reported an approximately 30% higher incidence among night-shift workers [32]. In a cross-sectional study of 53,053 Spanish workers, shift work was independently associated with higher surrogate insulin-resistance indices [33]. Direct glucose studies are smaller. A crossover study in 12 healthy nurses found higher postprandial glycemic excursion and reduced beta-cell responsivity during a simulated night shift [36]. In non-diabetic male workers, shift work was associated with higher nighttime mean glucose and 03:00 glucose, although several overall variability indices were similar in those with normal glucose regulation [35]. In female nurses, high-glycemic-index night-shift meals increased glucose concentration and GV, whereas meal frequency alone did not [37]. Among 37 healthcare shift workers with type 2 diabetes, mean glucose, MAGE, and CV did not differ across work conditions, although other variability metrics were higher on night shifts than on rest days after a night shift [38]. Circadian misalignment studies provide additional mechanistic support but do not substitute for direct occupational CGM evidence [39].

Sleep loss and poor sleep quality are associated with impaired insulin sensitivity and metabolic risk [40]. Whether these exposures consistently increase CGM-derived GV during real work schedules remains uncertain.

Psychosocial stress may affect glucose regulation through cortisol, catecholamines, and autonomic pathways [41]. The mechanism is plausible, but direct evidence for workday GV remains limited.

Physical activity usually improves insulin sensitivity and postprandial glucose handling, whereas intense or irregular activity can have different effects depending on diabetes status, insulin or medication use, meals, hydration, and counter-regulatory hormones [42].

### 5.3. Implications for Occupational Health Surveillance

The integration of glycemic variability into occupational health assessment may provide additional insights into metabolic risk among workers (Table 5). Traditional screening methods based solely on fasting glucose or HbA1c measurements may fail to identify individuals experiencing significant glucose fluctuations during daily activities.

Continuous glucose monitoring enables the evaluation of glucose dynamics during work shifts, sleep periods, meals, and physical activity. Direct studies in non-diabetic shift workers, healthcare workers, night-shift nurses, workers with type 2 diabetes, and a pilot worksite program have reported work- or meal-related differences in glucose profiles, but the evidence remains limited and heterogeneous [35,36,37,38,43,44]. Potential applications are summarized in Table 6.

For workers with known diabetes, CGM may support personalized clinical management when work schedules or safety-sensitive tasks create specific glycemic challenges. The diagnosis itself should never be treated as evidence of unfitness; assessment should focus on severe hypo-/hyperglycemia risk, treatment, awareness, monitoring, and individualized control [9,10,30].

For workers without diabetes, abnormal CGM patterns may be difficult to interpret and do not necessarily indicate disease. Confirmatory clinical assessment is required before diagnostic or employment-related conclusions are considered.

Broader workplace prevention should prioritize scheduling practices, healthy meal access, sleep-health programs, stress reduction, opportunities for physical activity, and diabetes education rather than relying primarily on monitoring.

From a preventive standpoint, identifying employees with elevated glycemic variability could help occupational physicians implement targeted interventions to improve metabolic balance. Such interventions might include lifestyle counseling, nutritional recommendations, optimizing work schedules, or encouraging consistent physical activity.

Furthermore, for workers already diagnosed with diabetes, CGM technologies may facilitate more personalized management strategies that account for work-related factors affecting glucose control. Monitoring glucose patterns during working hours could help reduce the risk of hypoglycemia or hyperglycemia in safety-critical settings.

Although continuous glucose monitoring is not yet common in workplace health assessments, its potential uses are worth exploring. Incorporating tools that measure metabolism into occupational health practices could help detect metabolic risks earlier. This would enable more personalized prevention strategies for employees.

Considering glycemic variability alongside traditional metabolic indicators could provide a more complete view of glucose regulation in working populations. This could ultimately support better worker health protection and risk management.

## 6. Practical Implications for Occupational Physicians

The increasing recognition of glycemic variability as a critical factor in glucose regulation implies several potential ramifications for occupational health protocols. Occupational physicians play a vital role in health surveillance programs aimed at preventing disease, identifying early risk factors, and ensuring the safe performance of job duties by employees. Therefore, a more comprehensive assessment of metabolic health could potentially improve both individual health outcomes and workplace safety measures.

Traditionally, occupational medicine has relied on measures like body mass index, lipid profiles, blood pressure, fasting glucose, and glycated hemoglobin to assess metabolic risk. These measures remain important for identifying individuals with impaired glucose metabolism or diabetes. However, as previously discussed, static glycemic markers might not fully capture the complex nature of glucose regulation during daily activities, including those related to work. Evaluating glycemic variability could provide additional insights in specific situations, especially for individuals whose metabolic balance may be affected by their work environment. For example, workers on rotating shifts, night shifts, or with irregular schedules may experience circadian misalignment, which could impact glucose metabolism. Similarly, employees under significant psychosocial stress or with irregular eating habits may show notable changes in their blood glucose levels throughout the workday. CGM technologies offer a potential method for assessing these dynamic metabolic responses. While routine CGM use is not currently recommended for general occupational health screenings, it could be justified in certain cases. These include research studies, targeted risk evaluations, or the care of employees with pre-existing diabetes working in safety-sensitive roles.

In selected workers with diabetes, CGM may help identify periods of increased hypoglycemia or hyperglycemia risk during work and support individualized treatment planning. Interpretation should follow diabetes-care guidance and remain under qualified clinical responsibility [9,10,30].

Occupational physicians can support preventive strategies focused on balanced nutrition, physical activity, sleep, and stress management. Workplace health-promotion programs may improve cardiometabolic risk factors, although outcomes depend on program design and implementation [45].

A broader view of glucose regulation, including its temporal changes, could help occupational health professionals spot early signs of metabolic changes. This would then allow them to create more targeted workplace prevention strategies.

## 7. Future Perspectives and Research Needs

Despite the growing interest in glycemic variability and its potential clinical implications, its application in occupational health remains relatively limited and requires further investigation. Most current evidence on glycemic variability comes from studies conducted in patients with diabetes or in hospital settings, whereas data specifically focused on working populations remain scarce. Expanding research in this field could help clarify how occupational exposures interact with metabolic regulation and influence glucose dynamics during daily activities.

Future studies should evaluate GV in clearly defined occupational groups using prospective cohorts, crossover comparisons of day and night shifts, and intervention designs. Longitudinal research is needed to determine whether CGM-derived measures add predictive value beyond established risk markers [47].

Future occupational studies may integrate CGM with actigraphy, sleep measures, dietary logs, and standardized exposure assessment. Such designs could clarify how work schedules, meal timing, sleep, and activity jointly influence glucose regulation while addressing feasibility and wearable-data limitations [31,46].

Standardizing glycemic variability metrics and their clinical interpretation is also a key research priority. Although several indices are available, consensus is still developing regarding which parameters are most informative and how they should be used outside traditional diabetes care.

Ultimately, more collaboration among occupational physicians, endocrinologists, epidemiologists, and biomedical engineers will be crucial to investigate the potential of glycemic variability as a new biomarker of metabolic risk in working populations.

## 8. Conclusions

This review has methodological limitations inherent to its narrative design. The search was targeted rather than systematic; study selection and synthesis were not performed in duplicate; and no formal risk-of-bias or certainty-of-evidence assessment was undertaken. Selective citation and publication bias therefore cannot be excluded.

The underlying evidence base is also limited. Direct occupational CGM studies are few, heterogeneous, and concentrated in shift workers, healthcare personnel, and selected worksite cohorts. Much of the mechanistic and prognostic evidence is extrapolated from diabetes-care, experimental, or hospital populations and may not generalize to metabolically healthy workers or different occupational systems. No validated GV thresholds exist for general workplace populations. Confounding by diet, sleep, chronotype, medications, physical activity, and baseline metabolic status; reverse causality; the healthy-worker effect; device-related measurement error; and differences in health-care systems constrain interpretation. Ethical, legal, cost, equity, and data-governance uncertainties further limit implementation. Glycemic control has traditionally been evaluated using static indicators such as fasting plasma glucose and glycated hemoglobin, which remain essential tools for diagnosing and monitoring metabolic disorders. However, growing scientific evidence indicates that these parameters may not fully capture the dynamic nature of glucose regulation. Glycemic variability represents an additional dimension of metabolic control that reflects the magnitude and frequency of glucose fluctuations over time and may contribute independently to oxidative stress, endothelial dysfunction, and vascular damage.

Advances in continuous glucose monitoring technologies have significantly improved the ability to evaluate glucose patterns in real-world settings. These devices offer detailed insights into glucose fluctuations during daily life, including work-related factors like shift schedules, sleep disturbances, psychosocial stress, and changes in physical activity. Although the use of glycemic variability assessment in occupational health is still developing, it could provide valuable opportunities to gain a better understanding of metabolic responses in the workplace.

Traditional markers such as fasting plasma glucose and HbA1c remain essential for identifying and monitoring dysglycemia, but they do not fully describe short-term glucose dynamics. GV and CGM-derived metrics may provide complementary information, particularly in selected diabetes-care contexts and in research examining work-related metabolic stressors. In occupational medicine, however, the evidence base remains early. Direct CGM studies in workers are limited, diabetes-care thresholds cannot be automatically applied to metabolically healthy workers, and ethical, legal, interpretive, and equity issues require careful attention. At present, CGM should not be presented as a tool for routine workplace screening. Its most defensible applications are the targeted clinical management of selected workers with diabetes, carefully governed fitness-for-work evaluations when clinically justified, intervention monitoring, and occupational research. Future studies should use standardized exposure assessment, pre-specified CGM protocols, adequate follow-up, and robust privacy safeguards to determine whether GV assessment can improve worker health protection without creating unintended harms.

For occupational physicians, integrating traditional metabolic markers with more dynamic indicators of glucose regulation may enhance the identification of early metabolic alterations and support more personalized prevention strategies. Further research is needed to clarify the role of glycemic variability in occupational health surveillance and determine whether its assessment can contribute to improved worker health protection and risk management. Ultimately, a more comprehensive evaluation of metabolic health may support safer workplaces and promote long-term well-being among workers. Future research integrating continuous glucose monitoring with occupational exposure assessment may provide novel insights into the interaction between work environment and metabolic health.

## Figures and Tables

**Table 1:** Limitations of static glycemic indicators and implications for occupational health. Abbreviations: FPG, fasting plasma glucose; GV, glycemic variability; HbA1c, glycated hemoglobin; OGTT, oral glucose tolerance test.

Marker	Key Limitation	Occupational Implication
FPG	Single fasting time point; affected by acute illness, sleep loss, stress, corticosteroids, and pre-analytical conditions.	May miss postprandial excursions and workday glucose fluctuations.
HbA1c	Reflects average exposure and is weighted toward recent weeks; affected by altered erythrocyte turnover, chronic kidney disease, hemoglobin variants, pregnancy, recent blood loss/transfusion, and iron deficiency.	May conceal hypoglycemia, hyperglycemic excursions, and high GV despite an apparently acceptable average value.
OGTT	Time-consuming, variably reproducible, and influenced by preparation, activity, illness, and stress.	Useful for impaired glucose tolerance but poorly suited to repeated occupational monitoring.
Single clinic measurements	Limited temporal resolution and ecological validity.	May not capture the effects of night work, irregular meals, workload changes, or sleep disruption.

**Table 2:** Common metrics used to assess glycemic variability. Main indices used to quantify glycemic variability in clinical and research settings. These metrics are commonly derived from continuous glucose monitoring data and provide complementary information about glucose dynamics. Abbreviations: CGM, continuous glucose monitoring; CV, coefficient of variation; HbA1c, glycated hemoglobin; MAGE, mean amplitude of glycemic excursions; SD, standard deviation; TAR, time above range; TBR, time below range; TIR, time in range.

Category	Metric	Operational Definition	Interpretation and Strengths	Caution for Occupational Use
Variability index	SD	Dispersion of glucose values around mean glucose.	Simple and widely reported.	Influenced by mean glucose, data completeness, and monitoring duration.
Variability index	CV	SD divided by mean glucose, expressed as a percentage.	Preferred relative variability index in diabetes-care CGM reports.	CV > 36% should not be generalized to non-diabetic workers or screening.
Variability index	MAGE	Mean amplitude of major glucose excursions.	Captures large swings and postprandial excursions.	Calculation-dependent, less intuitive, and sensitive to data quality.
CGM exposure metric	TIR	Time in a predefined target range, often 70–180 mg/dL in many adult diabetes-care contexts.	Clinically intuitive and complementary to HbA1c in diabetes care.	Target ranges may differ in pregnancy, prediabetes, healthy workers, and safety-sensitive contexts.
CGM exposure metric	TAR/TBR	Time above or below predefined ranges.	Summarizes hyperglycemia or hypoglycemia exposure.	Not pure variability metrics; thresholds require clinical justification.

**Table 4:** Occupational factors potentially influencing glucose regulation. GV, glycemic variability. Evidence categories distinguish direct CGM evidence from mechanistic or longer-term metabolic-risk evidence.

Occupational Factor	Mechanistic Pathway	Possible Glucose Effect	Evidence Appraisal	Representative Citations
Shift/night work	Circadian misalignment; altered insulin secretion, hormonal rhythms, sleep, and eating behavior.	Higher postprandial or nighttime glucose; possible changes in selected GV metrics.	Established association with diabetes risk; direct CGM evidence remains limited and heterogeneous.	[32,33,35,36,37,38,39]
Sleep disruption	Reduced insulin sensitivity, altered appetite hormones, sympathetic activation.	Higher mean glucose or excursions; effect on GV uncertain.	Experimental and epidemiological evidence; sparse occupational CGM data.	[40]
Psychosocial stress	Hypothalamic–pituitary–adrenal and sympathetic activation.	Transient glucose elevation; direct effect on occupational GV not established.	Mechanistically plausible; direct worker-CGM evidence limited.	[41]
Irregular meal timing	Circadian mistiming and altered postprandial metabolism.	Timing-dependent postprandial excursions.	Some direct intervention and observational evidence in night-shift workers.	[37,38,39]
Physical workload	Changes in muscle glucose uptake; effects depend on intensity, meals, hydration, medication, and diabetes status.	Acute glucose reduction or, with intense activity/stress, transient elevation.	Strong exercise physiology evidence; limited occupation-specific CGM data.	[42]
Sedentary work	Lower energy expenditure and muscle glucose utilization.	Higher postprandial exposure and insulin resistance over time.	Consistent metabolic-risk evidence; direct occupational GV evidence is sparse.	[33,42]

**Table 5:** Selected occupational studies evaluating glucose regulation or CGM in workers. Abbreviations: CGM, continuous glucose monitoring; CONGA, continuous overlapping net glycemic action; CV, coefficient of variation; GI, glycemic index; GV, glycemic variability; LDL, low-density lipoprotein; MAG, mean absolute glucose change; MAGE, mean amplitude of glycemic excursions.

Study	Population/Design	Monitoring or Intervention	Main Finding	Key Limitation
Ye et al., 2020 [35]	450 non-diabetic men; 238 shift workers; cross-sectional.	150 participants completed CGM for 3–7 days.	Shift workers had higher nighttime mean and 03:00 glucose; several overall variability indices were similar in normal glucose regulation.	Male-only, cross-sectional sample; limited causal inference.
Sharma et al., 2017 [36]	12 healthy nurses; randomized crossover simulated day vs. night shift.	Isotope-labeled mixed-meal testing with modeling of glucose flux and beta-cell function.	Night shift increased postprandial glycemic excursion and reduced beta-cell responsivity.	Small mechanistic study; simulated conditions.
Suyoto et al., 2024 [37]	Female nurses working real night shifts; randomized crossover.	Night-shift meals differing in glycemic index and frequency, assessed with CGM.	High-GI meals increased glucose concentration and GV; meal frequency alone was not the main determinant.	Short intervention; specific meal protocol and population.
Gibson et al., 2026 [38]	37 healthcare shift workers with type 2 diabetes; 10-day within-person study.	Blinded CGM, activity trackers, and diet/sleep diaries.	No difference in mean glucose, MAGE, or CV; MAG and CONGA were higher on night shifts than rest-after-night days.	Small, predominantly female healthcare sample.
Ko et al., 2025 [43]	234 workplace participants with type 2 diabetes or prediabetes; 8-week prospective cohort.	Two weeks of CGM plus personalized structured education.	In the type 2 diabetes group, HbA1c, fasting glucose, weight, and LDL cholesterol improved.	No randomized control; intervention combined CGM with education.
Crawford et al., 2021 [44]	207 employees or dependents at one worksite health center.	10-day blinded CGM plus HbA1c screening.	Feasible and acceptable; identified previously unrecognized diabetes and prediabetes and generated personalized profiles.	Single worksite; selected population and industry conflicts disclosed.

**Table 6:** Potential applications of continuous glucose monitoring technologies in occupational medicine and worker health surveillance.

Application Context	Purpose	Potential Value	Required Safeguards/Limitations
Research in shift workers	Assess glucose patterns across day, night, and rest days.	Clarifies exposure–response relationships and mechanisms.	Pre-specified protocols; adjustment for diet, activity, medication, sleep, and chronotype [35,36,37,38].
Work-activity studies	Assess responses during physically demanding or irregular tasks.	Improves understanding of task-related glucose dynamics.	Interpretation must account for diabetes status, treatment, meals, and workload [35,36,37,38,42].
Clinical management of workers with diabetes	Identify periods of hypo-/hyperglycemia risk during working hours.	Supports individualized management in selected safety-sensitive roles.	Clinical indication, qualified interpretation, confidentiality, and referral; diagnosis alone does not imply unsafe work [9,10].
Workplace health-promotion pilots	Evaluate diet, activity, or education programs.	Objective feedback and intervention evaluation.	Voluntary participation; no employer access to identifiable traces; avoid overdiagnosis [43,44,45].
Occupational epidemiology	Study determinants of metabolic health in real-world settings.	Links exposure assessment with glucose dynamics.	Ethics approval, data minimization, secure ownership, adequate sample size, and longitudinal design [35,36,37,38,43,44,46].

## Data Availability

No new data were created or analyzed in this study.

## References

[1] International Diabetes Federation (2025). IDF Diabetes Atlas.

[2] American Diabetes Association Professional Practice Committee for Diabetes (2026). 2. Diagnosis and Classification of Diabetes: Standards of Care in Diabetes—2026. Diabetes Care.

[3] World Health Organization (2019). Classification of Diabetes Mellitus.

[4] Monnier L., Colette C. (2008). Glycemic variability: Should we and can we prevent it?. Diabetes Care.

[5] Monnier L., Mas E., Ginet C., Michel F., Villon L., Cristol J.-P., Colette C. (2006). Activation of oxidative stress by acute glucose fluctuations compared with sustained chronic hyperglycemia in patients with type 2 diabetes. JAMA.

[6] Brownlee M. (2005). The pathobiology of diabetic complications: A unifying mechanism. Diabetes.

[7] Ceriello A., Esposito K., Piconi L., Ihnat M.A., Thorpe J.E., Testa R., Boemi M., Giugliano D. (2008). Oscillating glucose is more deleterious to endothelial function and oxidative stress than mean glucose in normal and type 2 diabetic patients. Diabetes.

[8] Battelino T., Danne T., Bergenstal R.M., Amiel S.A., Beck R., Biester T., Bosi E., Buckingham B.A., Cefalu W.T., Close K.L. (2019). Clinical targets for continuous glucose monitoring data interpretation: Recommendations from the International Consensus on Time in Range. Diabetes Care.

[9] Danne T., Nimri R., Battelino T., Bergenstal R.M., Close K.L., DeVries J.H., Garg S., Heinemann L., Hirsch I., Amiel S.A. (2017). International Consensus on Use of Continuous Glucose Monitoring. Diabetes Care.

[10] American Diabetes Association Professional Practice Committee for Diabetes (2026). 7. Diabetes Technology: Standards of Care in Diabetes—2026. Diabetes Care.

[11] Nathan D.M., Kuenen J., Borg R., Zheng H., Schoenfeld D., Heine R.J. (2008). Translating the A1C assay into estimated average glucose values. Diabetes Care.

[12] Stratton I.M., Adler A.I., Neil H.A.W., Matthews D.R., Manley S.E., Cull C.A., Hadden D., Turner R.C., Holman R.R. (2000). Association of glycaemia with macrovascular and microvascular complications of type 2 diabetes (UKPDS 35): Prospective observational study. BMJ.

[13] Service F.J. (2013). Glucose variability. Diabetes.

[14] Little R.R., Sacks D.B. (2009). HbA1c: How do we measure it and what does it mean?. Curr. Opin. Endocrinol. Diabetes Obes..

[15] Ceriello A., Ihnat M.A. (2010). “Glycaemic variability”: A new therapeutic challenge in diabetes and the critical care setting. Diabet. Med..

[16] Lamprou S., Evangelidis N., Koletsos N., Zografou I., Stoimeni A., Mintziori G., Gkolias V., Trakatelli C.-M., Savopoulos C., Doumas M. (2026). Microvascular Dysfunction in Patients with Prediabetes: Novel Methods Identify Impaired Microcirculation. Life.

[17] Lamprou S., Koletsos N., Zografou I., Lazaridis A., Mintziori G., Trakatelli C.-M., Kotsis V., Gkaliagkousi E., Doumas M., Triantafyllou A. (2024). Skin Microvascular Dysfunction in Type 2 Diabetes Mellitus Using Laser Speckle Contrast Analysis and Association with Carotid Intima-Media Thickness. J. Clin. Med..

[18] DeFronzo R.A. (2004). Pathogenesis of type 2 diabetes mellitus. Med. Clin. N. Am..

[19] Woerle H.J., Neumann C., Zschau S., Tenner S., Irsigler A., Schirra J., Gerich J.E., Göke B. (2007). Impact of fasting and postprandial glycemia on overall glycemic control in type 2 diabetes: Importance of postprandial glycemia to achieve target HbA1c levels. Diabetes Res. Clin. Pract..

[20] Rodbard D. (2009). Interpretation of continuous glucose monitoring data: Glycemic variability and quality of glycemic control. Diabetes Technol. Ther..

[21] Service F.J., Molnar G.D., Rosevear J.W., Ackerman E., Gatewood L.C., Taylor W.F. (1970). Mean amplitude of glycemic excursions, a measure of diabetic instability. Diabetes.

[22] Beck R.W., Bergenstal R.M., Riddlesworth T.D., Kollman C., Li Z., Brown A.S., Close K.L. (2019). Validation of Time in Range as an Outcome Measure for Diabetes Clinical Trials. Diabetes Care.

[23] Egi M., Bellomo R., Stachowski E., French C.J., Hart G. (2006). Variability of blood glucose concentration and short-term mortality in critically ill patients. Anesthesiology.

[24] Hirsch I.B., Brownlee M. (2005). Should minimal blood glucose variability become the gold standard of glycemic control?. J. Diabetes Complicat..

[25] Didyuk O., Econom N., Guardia A., Livingston K., Klueh U. (2021). Continuous Glucose Monitoring Devices: Past, Present, and Future Focus on the History and Evolution of Technological Innovation. J. Diabetes Sci. Technol..

[26] Beck R.W., Riddlesworth T., Ruedy K., Ahmann A., Bergenstal R., Haller S., Kollman C., Kruger D., McGill J.B., Polonsky W. (2017). Effect of Continuous Glucose Monitoring on Glycemic Control in Adults with Type 1 Diabetes Using Insulin Injections: The DIAMOND Randomized Clinical Trial. JAMA.

[27] Ajjan R.A., Cummings M.H., Jennings P., Leelarathna L., Rayman G., Wilmot E.G. (2019). Optimising use of rate-of-change trend arrows for insulin dosing decisions using the FreeStyle Libre flash glucose monitoring system. Diabetes Vasc. Dis. Res..

[28] Bandodkar A.J., Wang J. (2014). Non-invasive wearable electrochemical sensors: A review. Trends Biotechnol..

[29] U.S. Food and Drug Administration (2024). Do Not Use Smartwatches or Smart Rings to Measure Blood Glucose Levels: FDA Safety Communication. https://www.fda.gov/medical-devices/safety-communications/do-not-use-smartwatches-or-smart-rings-measure-blood-glucose-levels-fda-safety-communication.

[30] Scharf J., Nguyen X.Q., Vu-Eickmann P., Krichbaum M., Loerbroks A. (2019). Perceived Usefulness of Continuous Glucose Monitoring Devices at the Workplace: Secondary Analysis of Data from a Qualitative Study. J. Diabetes Sci. Technol..

[31] Piwek L., Ellis D.A., Andrews S., Joinson A. (2016). The Rise of Consumer Health Wearables: Promises and Barriers. PLoS Med..

[32] Xie F., Hu K., Fu R., Zhang Y., Xiao K., Tu J. (2024). Association between night shift work and the risk of type 2 diabetes mellitus: A cohort-based meta-analysis. BMC Endocr. Disord..

[33] Tosoratto J., Tárraga López P.J., López-González Á.A., Busquets-Cortes C., Obrador de Hevia J., Ramirez-Manent J.I. (2025). Associations Between Shift Work and Insulin Resistance Markers in 53,053 Spanish Workers: A Sex-Stratified Cross-Sectional Analysis Using TyG, TyG-BMI, METS-IR, and SPISE-IR Indices. J. Clin. Med..

[34] Li A.K., Nowrouzi-Kia B. (2017). Impact of Diabetes Mellitus on Occupational Health Outcomes in Canada. Int. J. Occup. Environ. Med..

[35] Ye L., Gu W., Chen Y., Li X., Shi J., Lv A., Hu J., Zhang R., Liu R., Hong J. (2020). The impact of shift work on glycemic characteristics assessed by CGM and its association with metabolic indices in non-diabetic subjects. Acta Diabetol..

[36] Sharma A., Laurenti M.C., Dalla Man C., Varghese R.T., Cobelli C., Rizza R.A., Matveyenko A., Vella A. (2017). Glucose metabolism during rotational shift-work in healthcare workers. Diabetologia.

[37] Suyoto P.S.T., de Rijk M.G., de Vries J.H.M., Feskens E.J.M. (2024). The Effect of Meal Glycemic Index and Meal Frequency on Glycemic Control and Variability in Female Nurses Working Night Shifts: A Two-Arm Randomized Cross-Over Trial. J. Nutr..

[38] Gibson R., D’Annibale M., Palla L., Tejo F., McGowan B., Vetter C., Oliver N., Lorencatto F., Guess N. (2026). Characterising the impact of shift work on diet and glucose variability in healthcare employees living with type 2 diabetes: The Shift-Diabetes Study. Diabet. Med..

[39] Morris C.J., Purvis T.E., Hu K., Scheer F.A.J.L. (2016). Circadian misalignment increases cardiovascular disease risk factors in humans. Proc. Natl. Acad. Sci. USA.

[40] Knutson K.L., Spiegel K., Penev P., Van Cauter E. (2007). The metabolic consequences of sleep deprivation. Sleep Med. Rev..

[41] Chrousos G.P. (2009). Stress and disorders of the stress system. Nat. Rev. Endocrinol..

[42] Colberg S.R., Sigal R.J., Yardley J.E., Riddell M.C., Dunstan D.W., Dempsey P.C., Horton E.S., Castorino K., Tate D.F. (2016). Physical Activity/Exercise and Diabetes: A Position Statement of the American Diabetes Association. Diabetes Care.

[43] Ko J.-H., Moon S.-J., Ajjan R.A., Lee M.Y., Lee H.-J., Choi B., Park J., Lee S.-E., Kang J.-H., Park C.-Y. (2025). Workplace-based continuous glucose monitoring with structured education for pre-diabetes and type 2 diabetes: A prospective community cohort study. Diabetes Obes. Metab..

[44] Crawford M., Johnson M., Klein I.J., Hames K.C., Norman G.J. (2021). Including Continuous Glucose Monitoring to Provide Personalized Glycemic Profiles as Part of a Pilot Worksite Health Screening. J. Diabetes Sci. Technol..

[45] Goetzel R.Z., Ozminkowski R.J. (2008). The health and cost benefits of work site health-promotion programs. Annu. Rev. Public Health.

[46] Alsaedi S., Skogstad M., Haugen F. (2025). GLU24/7 study: Cardiometabolic health risk factors in night shift workers—Protocol for a 2-year longitudinal study in an industrial setting in Norway. BMJ Open.

[47] Tabák A.G., Herder C., Rathmann W., Brunner E.J., Kivimäki M. (2012). Prediabetes: A high-risk state for diabetes development. Lancet.

